# Maximum Running Speed of Captive Bar-Headed Geese Is Unaffected by Severe Hypoxia

**DOI:** 10.1371/journal.pone.0094015

**Published:** 2014-04-07

**Authors:** Lucy A. Hawkes, Patrick J. Butler, Peter B. Frappell, Jessica U. Meir, William K. Milsom, Graham R. Scott, Charles M. Bishop

**Affiliations:** 1 School of Biological Sciences, Bangor University, Bangor, Gwynedd, United Kingdom; 2 School of Biosciences, University of Birmingham, Birmingham, United Kingdom; 3 University of Tasmania, Hobart, Tasmania, Australia; 4 Department of Anesthesia, Critical care and Pain Medicine, Harvard Medical School, Massachusetts General Hospital, Boston, Massachusetts, United States of America; 5 Department of Zoology, University of British Columbia, Vancouver, Canada; 6 Department of Biology, McMaster University, Hamilton, Ontario, Canada; 7 University of Exeter, College of Life and Environmental Sciences, Penryn Campus, Penryn, Cornwall, United Kingdom; James Cook University, Australia

## Abstract

While bar-headed geese are renowned for migration at high altitude over the Himalayas, previous work on captive birds suggested that these geese are unable to maintain rates of oxygen consumption while running in severely hypoxic conditions. To investigate this paradox, we re-examined the running performance and heart rates of bar-headed geese and barnacle geese (a low altitude species) during exercise in hypoxia. Bar-headed geese (n = 7) were able to run at maximum speeds (determined in normoxia) for 15 minutes in severe hypoxia (7% O_2_; simulating the hypoxia at 8500 m) with mean heart rates of 466±8 beats min^−1^. Barnacle geese (n = 10), on the other hand, were unable to complete similar trials in severe hypoxia and their mean heart rate (316 beats.min^−1^) was significantly lower than bar-headed geese. In bar-headed geese, partial pressures of oxygen and carbon dioxide in both arterial and mixed venous blood were significantly lower during hypoxia than normoxia, both at rest and while running. However, measurements of blood lactate in bar-headed geese suggested that anaerobic metabolism was not a major energy source during running in hypoxia. We combined these data with values taken from the literature to estimate (i) oxygen supply, using the Fick equation and (ii) oxygen demand using aerodynamic theory for bar-headed geese flying aerobically, and under their own power, at altitude. This analysis predicts that the maximum altitude at which geese can transport enough oxygen to fly without environmental assistance ranges from 6,800 m to 8,900 m altitude, depending on the parameters used in the model but that such flights should be rare.

## Introduction

Bar-headed geese (*Anser indicus*) and barnacle geese (*Branta leucopsis*) undertake similar long distance migratory flights between breeding and wintering grounds, usually covering thousands of kilometres, during the autumn and spring [1], [2]. Bar-headed geese travel over 5,000 km from Indian wintering grounds and high Asian breeding grounds in China and Mongolia [2], [3], crossing the Himalayan mountains (mean elevation 4,500 metres) en route. Barnacle geese travel up to 2,500 km from wintering grounds in the UK [4]–[8] to high arctic breeding grounds (e.g. Svalbard), and have been shown to travel much of the way over the ocean and at sea level. The cardiovascular and muscular adaptations of both species must be sufficient to maintain full aerobic metabolism during such sustained long distance flight but the enormous altitudinal differences between the migrations undertaken by the two species raises some interesting questions. Bar-headed geese must typically fly at over 4,000 m elevation for many hundreds of kilometres while traversing the Himalayan mountains and Tibetan Plateau [2]. At such high altitudes, barometric pressure is substantially reduced along with the partial pressure of oxygen (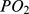), leading to progressive environmental hypoxia and, therefore, a decrease in the oxygen available to fuel flapping flight. Barnacle geese, flying at sea level, are not exposed to such a challenge. Sustaining any form of exercise when at high altitude is extremely challenging but bar-headed geese have been shown to possess a number of specific physiological adaptations that may increase their performance, relative to other species of geese when exposed to severe environmental hypoxia (reviewed in [9]–[11]). Studies on captive birds have demonstrated that bar-headed geese at rest have remarkable hypoxia tolerance compared to other similar sized waterfowl [12]–[15], including barnacle geese, and even remain conscious and upright at an inspired 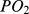 equivalent to that at an altitude of 12,190 metres [16].

Despite the superior ability of bar-headed geese to tolerate severe hypoxia at rest, it is less clear whether this is true of these birds during exercise. Direct comparisons between similarly sized barnacle and bar-headed geese while running on a treadmill or flying in a wind tunnel in normoxia show little difference in the relationship between cardiac frequency and the rate of oxygen consumption [17]. This indicates that the flight physiology and biomechanics of these two species may be comparable during near sea-level flight. In the only study of bar-headed geese exercising while exposed to severe hypoxia (7% oxygen), Fedde et al. [18] reported that bar-headed geese running on a treadmill did not increase the rate of oxygen consumption relative to that at rest, suggesting that delivery of oxygen to the working muscles may have been significantly limited. In contrast, the level of CO_2_ production approximately doubled during exercise in both hypoxia and normoxia. Thus, the work by Fedde et al. [18] suggested, paradoxically, that the estimated value for cardiac output was unchanged between resting and exercise in hypoxia and could not explain how the additional cost of exercise could be met. The birds in Fedde et al. [18] were instrumented with face masks, however, and these may have inhibited their ability to perform if the experimental conditions were excessively stressful [19], as suggested by the unexpectedly high values for heart rate recorded during both resting and running exercise in normoxia. Meanwhile, the ability of barnacle geese to tolerate environmental hypoxia during exercise has never been tested. In the present study, therefore, we set out to revisit the work by Fedde [18] to understand how the costs of exercise might be met by bar-headed geese, and to compare their performance with barnacle geese during exercise while exposed to environmental hypoxia.

Bar-headed geese are renowned as one of the world's highest flying migrants and have been reported to migrate regularly at extremely high altitudes (>8,000 m), while crossing the ridges and summits of some of the highest peaks of the Himalayas [20], [21], where conditions would be extremely hypoxic. However, this hypothesis arises only from anecdotal accounts [20], [22] and the highest direct measurement of flight altitude from satellite telemetry is 7290 m [2], [3]. Therefore, in the present study, we set out to model the maximum altitude at which bar-headed geese might be found in flight, using cardiovascular and respiratory performance from the literature during running exercise as input variables, with appropriate caveats.

## Aims

In the present study we aimed to: (i) compare the overall performance of bar-headed geese, a high altitude specialist, to that of the similar but lowland migrating barnacle geese under two hypoxic conditions: the first at inspired 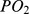 equivalent to 5,500 metres, similar to the Tibetan Plateau, and the second at inspired 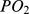 equivalent to 8,500 metres, nearly the summit of Mount Everest. At these altitudes, atmospheric pressures (and thus 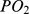) are a half and one third the sea level value, respectively. Secondly, (ii) we aimed to compare our data with previous work by Fedde et al. [18] and; (iii) to use measured values for blood gases of bar-headed geese to model an estimate for the threshold maximum altitude for aerobic flight by wild bar-headed geese.

## Methods

### Experimental animals

Twelve bar-headed geese and twelve barnacle geese were obtained as newly fledged birds from captive collections in the UK. All the geese were hatched near sea level and pinioned before five weeks of age. The birds had therefore never flown nor experienced high altitude. They were kept in the University of Birmingham outdoor aviary and fed with mixed grain and provided with clean running water. The geese were trained to run on a variable speed treadmill (SportsArt 1098F) inside a perspex respirometer box (see below) in normoxia, and the highest running speed (without incline) that each goose could maintain for 15 minutes was determined by repeating trials at increasing treadmill speeds. Geese were generally trained for several hours a day, with at least one day of rest between training days, until the maximum sustainable running speed was determined. Training took place over several weeks due to the number of study individuals and was repeated at intervals of several months throughout the data collection period. Maximum sustainable speed was confirmed for each goose when it was unable to complete the 15-minute trial at a faster speed (either failing to maintain station in the centre of the treadmill belt, tripping up repeatedly or refusing to run). The treadmill speeds used are the fastest reported to date for this species [17] and it was not possible to persuade geese to run any faster. The individual maximum running speeds were applied to all subsequent experiments for each goose. Five bar-headed geese and two barnacle geese were unable to run consistently on the treadmill and were excluded from the exercise experiments but were used for measurements at rest. The mean body mass of the 7 remaining bar-headed geese was 2.33 kg±0.05 s.e.m., while mean body mass for the 10 remaining barnacle geese was 1.79 kg±0.09 s.e.m.. In each experiment, geese walked on the treadmill at a warm up speed of 0.44 metres per second (m.s^−1^) for 5 minutes, then ran for 15 minutes at their individual maximum running speed. The treadmill was stopped as soon as any individual stopped running or could not run consistently so that the birds were never run to exhaustion.

### Heart rate

Geese were fitted with subcutaneous gold-plated pin electrodes connected to an AD Instruments BioAmp unit, which collected continuous electrocardiogram (ECG), to measure heart rate (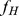) during experiments. In addition, custom-built heart rate data loggers were implanted into the abdominal cavity of bar-headed geese under general anaesthesia (2 to 4% isofluorane in medical grade oxygen mixture; (see also [4], [24]). The loggers recorded continuous ECG, core body temperature and three-axis accelerometry for four months. Geese were given a minimum of two weeks to recover from the surgery before being included in experiments. Heart rate was measured for a representative sub-section of the total recording, usually consisting of the last two to three minutes of each resting or exercising bout, and counted by eye over 10 second intervals. Heart rate was also derived from the data loggers using custom script in Delphi (www.borland.com).

### Respirometry

Rates of oxygen consumption (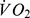) and carbon dioxide production (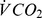) were measured by placing a goose inside the respirometer (60×60×48 cm; volume 173 L). The respirometer rested on a wooden frame that was sealed at the sides to the treadmill with closed cell compressible rubber, and clamped into position. The sides of the respirometer were covered to reduce disturbance of the geese during the experimental runs, and the rear was obscured with a removable barrier during resting phases. Gas was pulled through the respirometer using pumps (Rietschle Thomas) running through a carboy to dampen pressure fluctuations, and flow measured with a thermal mass flow meter (Bronkhorst Mass View MV106). Measured flow rate was in the range of 56 to 144 L min^−1^, varying between experiments for practical purposes. Air entered the respirometer through the small gaps at the front and back of the treadmill belt. The air in the respirometer was mixed using a single 12 V fan in a side compartment (RS components) and air leaks were minimised using draft excluder brushes at the front and rear of the respirometer where the treadmill belt was positioned. Although all efforts were made to minimise air leaks into the respirometer, which would have caused additional air to be drawn into the respirometer, decreasing measured 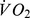 and 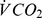, they were tested by flowing oxygen-free nitrogen at a known rate into the respirometer at the different treadmill speeds used in the experiments [25]. Leaks could be determined as the difference between the observed and expected change in O_2_ in the respirometer. Resultant leak correction values were applied to the experimental data following Fedak et al. [25].

**Figure 1:**
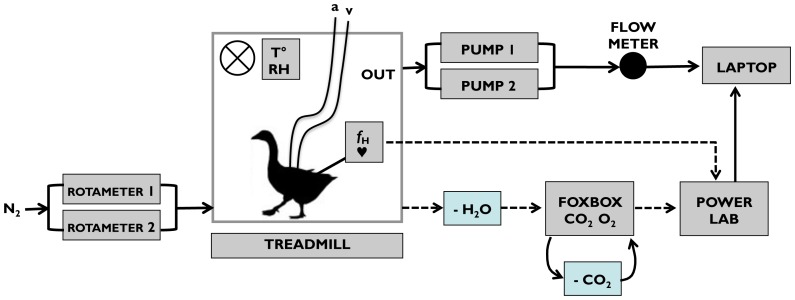
Experiment set up for bar-headed goose respirometry. Heart rate (*f*_H_) was recorded both externally using an ADI BioAmp with subcutaneous electrodes and internally using custom made loggers (see methods). Temperature (T°) and relative humidity (RH) were measured inside the respirometer and used to correct flow rate through the respirometer to STPD conditions (see methods). Subsamples of well-mixed air from the respirometer (fan indicated as cross in circle) were first dried (- H_2_O) before analysis of fractional concentration of CO_2_ and O_2_ in a gas analyser. Analyser data was passed via a PowerLab to a computer. To create conditions of hypoxia, appropriate amounts of N_2_ were flowed into the respirometer. Arterial (a) and mixed venous (v) blood samples were taken through cannulae exiting the lid of the respirometer.

Three experimental respirometry settings were used: (i) normoxia (20.95% O_2_); (ii) moderate hypoxia (10.5% O_2_, simulating 5,500 m altitude in the international standard atmosphere) and (iii) severe hypoxia (7% O_2_, simulating 8,500 m altitude, approximating the top of Mount Everest at 8,848 m). The order in which experiments were carried out were standardised: values were first recorded for geese at rest and exercising in normoxia, then the experiment was repeated in moderate and then severe hypoxia. Geese were never exposed to more than one hypoxic treatment on any given day. Conditions of hypoxia were obtained in the respirometer by flowing appropriate rates of O_2_-free N_2_ to displace half and two-thirds of the O_2_ respectively (Fig. 1). Subsamples of respirometer gas at 200 mL.min^−1^ were pulled first through a drying column (Drierite, Hammond) and then through a Sable Systems FoxBox, or AD Instruments gas analyser, by an internal pump in the analyser. The outputs from these instruments were processed and analysed using a 16-channel PowerLab and ADI Chart software (Fig. 1). The gas analyser was zeroed and calibrated before and after each experiment using O_2_ free N_2_, a 5% O_2_ mix in O_2_-free N_2_, and outside air [25].

Rates of oxygen consumption (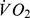) were calculated as the difference between fractional concentrations of O_2_ in dry incurrent (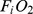) and excurrent (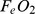) air, where CO_2_ is not scrubbed from the system [26], [27]:
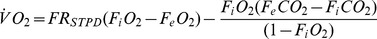
(1)


Where 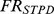 is excurrent flow rate corrected to standard dry temperature and pressure conditions (273 °K = 0°C) and 101.3 kPa, where 1 mole of gas occupies 22.4 litres. Rate of CO_2_ production (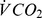) was calculated as:
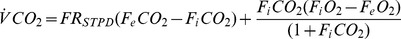
(2)


Prior to each running experiment, geese rested in the respirometer for at least one hour after which ‘
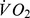
at rest’ and ‘heart rate at rest’ were recorded.

It was not possible to measure 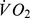 accurately when the birds were exposed to hypoxia as it proved impossible to repeatedly produce an accurately known incurrent baseline value of O_2,_ with values varying by up to 0.2% in calibrations of the respirometer before experiments took place. 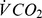 could be measured in hypoxia, however, because baseline incurrent values were stable at <0.01% CO_2_. This meant that we were able to measure 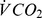 at rest and during exercise in normoxia and hypoxia but we could only measure 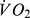 at rest and during exercise in normoxia. We, therefore, estimated 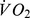 values for birds at rest and during exercise in hypoxia using the respiratory exchange ratio (RER, which is equivalent to respiratory quotient - RQ - under steady-state conditions) values derived for the present study of bar-headed geese at rest and during exercise, which were the same (0.81). Importantly, this may have over estimated 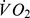 if RER was higher, which might be expected during hyperventilation, acidosis (for example during lactate build-up) and if there were an increased preference for carbohydrate oxidation during hypoxia [28], although this depends on whether animals had reached a steady state. We highlight that these values are an estimate and are to be interpreted cautiously.

### Blood gases

In order to collect blood gas variables from bar-headed geese, heparinised cannulae (sterilised PE90 tubing filled with heparinised saline at 20 IU.mL^−1^) were placed in the brachial artery and vein of the right wing under general anaesthesia [29]. The vessels were blunt dissected free of the fascia, ligated on the distal side and secured on the proximal side using a vascular clamp. A small cut was made in the vessel, and the cannula introduced. The venous cannula was inserted 8.5 cm into the brachial vein (terminating in the superior vena cava) to sample mixed-venous blood and the location confirmed post-mortem. The arterial cannula was introduced 5.2 cm into the brachial artery. Both cannulae were anchored in place with suture behind a ‘bulb’ in the PE90 (prepared before introduction). We did not cannulate the leg vein because Fedde et al. [18] found no significant difference in blood gases or pH between mixed venous and leg vein blood. Experiments were carried out in normoxia and in severe hypoxia (7% O_2_) but not in moderate hypoxia (10.5% O_2_) since bar-headed geese could manage running in severe hypoxia. The order in which the two experiments, normoxia and severe hypoxia, were carried out was randomised to reduce any possible effects of haematocrit reduction (through sampling) on performance. Blood gases were not measured in barnacle geese.

Arterial and mixed venous blood samples (<1.5 mL each) were drawn after the goose had been at rest for one hour in the respirometer before the beginning of each experiment. Sample size (representing approximately 0.6% of total blood volume for a 2.33 kg goose; [30]) was required to provide sufficient samples and replicates for analyses using multiple pieces of equipment (i.e. not using a single automated blood gas analyser, which requires very small blood volumes). Arterial and mixed venous blood samples were taken six minutes into each experiment, when the bird was running at a steady pace and 99% of the air volume in the respirometer had been replaced. This should have meant that the percent O_2_ in the respirometer would have been stable. The cannulae were refilled after each sampling event with heparinised saline (10 IU.mL^−1^) to prevent clotting in the line. Partial pressures of O_2_ (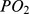) and CO_2_ (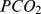) were measured using glass electrodes (Loligo systems, Denmark) connected to a Radiometer PHM73 (Radiometer Limited, UK). The 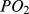 electrode was zeroed using saturated sodium sulphite solution and spanned using air saturated water. The 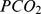 electrode was spanned using water saturated with custom 2% and 5% CO_2_ gas mixtures (British Oxygen Cylinders).

The oxygen content of 10 μl samples of blood was determined for both arterial (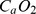) and mixed venous (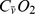) blood using the method described by Tucker (35). The O_2_ electrode was zeroed daily using sodium sulphite and spanned after each set of measurements using aerated water. After each sample the sample chamber was rinsed with deionized water. Blood lactate concentration was measured using an Accutrend plus meter (Roche Products Limited, UK) and haemoglobin concentration was measured using an Hb201+ meter (HemoCue Inc., USA; which uses a modified azidemethemoglobin reaction, accurate to within ±5% for avian blood absorbance values [31]). We also determined haematocrit of the arterial and mixed venous blood by centrifugation of capillary tubes of each sample.

Using measured heart rate, 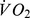, 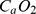 and 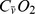, we, used the Fick equation (Equation 3, below) for the convection of blood to estimate stroke volume (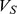). Cardiac output (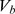) was then calculated by multiplying the estimated stroke volumes by the recorded heart rates.

### Ventilation frequency

In a subset of experiments, four bar-headed geese were fitted with nasal thermistors to measure the difference in respiratory frequency (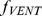) between normoxia and hypoxia. While we could measure ventilation frequency in this manner in birds prior to and during exercise, it was not possible to collect 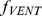 from geese that were rested for long periods to obtain minimum ventilation frequency, as the animals would dislodge the thermistor.

Experiments on the bar-headed geese were structured to take into account the increasing effect of instrumentation on the birds. i) Experiments were first carried out on geese with only subcutaneous ECG electrodes; (ii) experiments were then repeated at least two weeks after implantation of heart rate data loggers (see above) and finally iii) experiments were repeated after geese were implanted with cannulae into an artery and a vein.

### Acceleration

Because it was not possible to measure 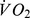 directly when the birds were running during exposure to hypoxia, we used three-axis accelerometer data to derive vectoral dynamic body acceleration (VeDBA, [32]) to compare the physical work carried out during experiments in normoxia and hypoxia. To calculate VeDBA, we calibrated each axis against gravity, subtracted the static acceleration using a running mean over 5 seconds (encompassing approximately 25 cycles of the behaviour of interest, in this case, footfalls) and took the square root of the sum of the three axes squared to derive mean VeDBA values.

### Modelling maximum flight altitude

Using blood gas values from the present study and supplementing them with published data, it should be possible to provide a baseline estimate for how the circulatory system of the bar-headed goose might support aerobic flight at altitude (Fig.S1). While the blood gas values in the present study are measured from geese during running, rather than flying (i.e. hind limb rather than fore limb exercise), the most important consideration for the present modelling approach is on the supply of oxygen by the cardiovascular system to the working muscles, rather than the ability of leg or flight muscles to actually utilise the oxygen supplied. Thus, unless there are fundamental differences between how the lung functions during running and flight, it is expected that realistic values for arterial oxygen loading should result. The model also assumes still air conditions, without opportunity for orographic lift or thermal updrafts and uses values for the international standard atmosphere (see also [2]).

The maximum rate of oxygen that can be conveyed by the circulation can be modelled using the Fick equation for the convection of blood:
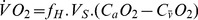
(3)


In this equation, the rate of oxygen consumption (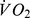) can be expressed as a function of heart rate (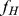), cardiac stroke volume (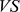) and the arterio-venous oxygen difference (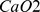 - 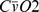). During high altitude flight, bar-headed geese can only fly at or below 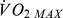 (i.e. the maximal rate of oxygen consumption), which can be modelled by inputting maximum expected values for terms on the right hand side of the equation. The maximum mean 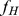 recorded during sustained exercise in the present study was 466 bpm, and an estimate of 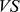 can be derived from the present study (but note that this could not be measured directly – see below). The maximum 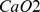 measured during exercise and a mean minimal value for 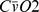 in the present study can then also be inputted to calculate the maximum rate at which bar-headed geese could supply oxygen to the muscles during flight at high altitude (and hence from here on referred to as ‘*supply*’).

Supply, however, is expected to decrease with increasing altitude as barometric pressure, and hence 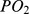, drop, meaning that blood oxygen saturation will be progressively reduced. We modelled supply at high altitude (*supply*_HA_) by constructing a haemoglobin-oxygen equilibrium curve from blood-gas values obtained in the present study and drawing the maximum expected haemoglobin saturation (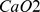
_)_ for different environmental 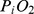, assuming that arterial 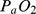
differs from inspired 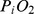
by only 5% [11]. At the same time, the aerodynamic power required for flight (from here on referred to as *demand*) is expected to increase with increasing altitude because air density begins to decline, meaning that proportionally more energy must be expended to stay aloft at a given velocity (28). We therefore used values for air density and temperature taken from the international standard atmosphere and flight aerodynamic theory [33] to model the proportional increase in oxygen demand for flight at high altitude (*demand*_HA_). We then multiplied 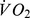 values measured for geese flying in a wind tunnel at sea level [17] by this additional factor. Finally, we derived the maximum predicted flight altitude as the point at which *supply* and *demand* cross over.

### Statistics

All values are presented as the grand mean of mean values per individual (± standard error of the mean, s.e.m.) to avoid pseudo-replication. Data were compared using paired t-tests and significance differences between two mean values assumed at 5%.

### Ethics

All work in this study was carried out by personnel in possession of a Home Office personal license under Project license 30/2711 awarded to PJB, as set out in the Animals (Scientific Procedures) Act 1986 of the UK and all efforts were taken to minimise animal suffering.

## Results

The maximum speeds that bar-headed geese (n = 7) and barnacle geese (n = 10) could sustain for 15 minutes in normoxia ranged from 0.96 to 1.17 m.s^−1^. The same speeds could also be sustained for 15 minutes by both species in moderate hypoxia (10.5% O_2_) with no signs of fatigue. Bar-headed geese also successfully sustained their maximum speeds in severe hypoxia (7% O_2_). Barnacle geese, however, could not sustain maximum speeds in severe hypoxia, managing no more than four minutes (see below).

### 1. Bar-headed geese

#### i) Heart rate

**Figure 2:**
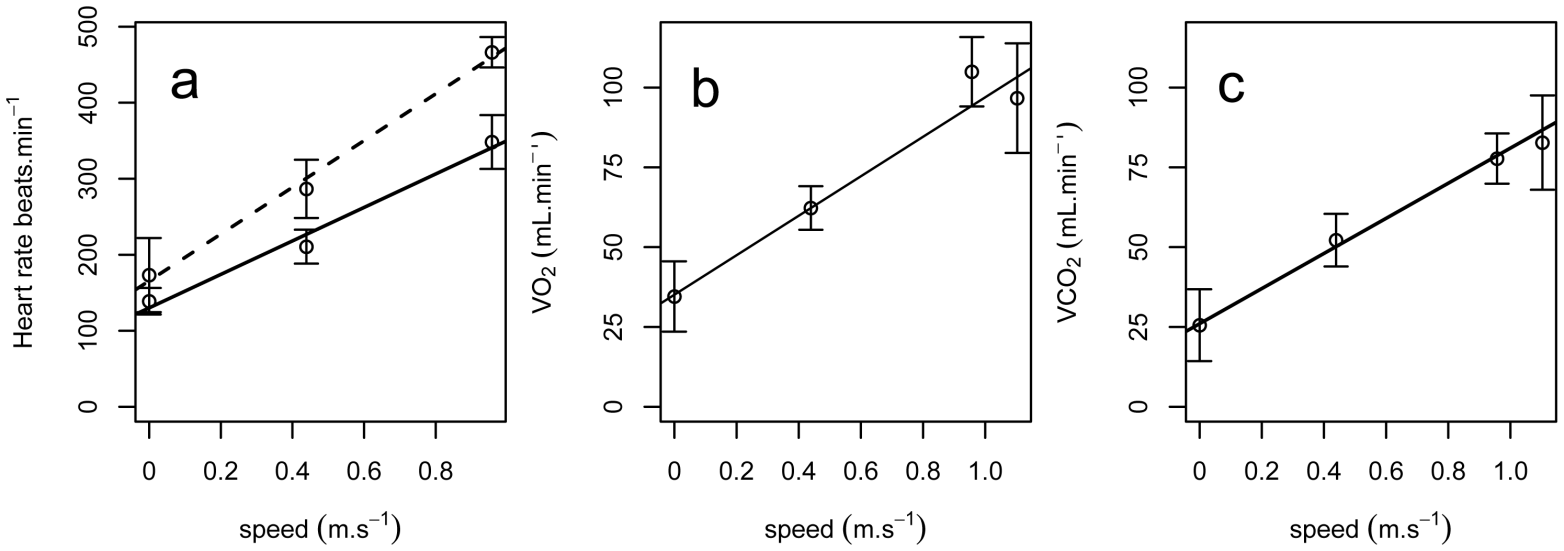
Heart rate and metabolic rate of bar-headed geese. (a) Mean heart rate, (b) Mean 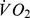 (in mL.min^−1^) and Mean 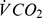 (in mL.min^−1^) of un-cannulated bar-headed geese against increasing treadmill speed in normoxia (solid line) and hypoxia (dashed line in part a), error bars show s.e.m.; Lines show linear models for fit, error bars show ±1 s.e.m.

When in normoxia, bar-headed geese with implanted data loggers and cannulae had a mean heart rate at rest, prior to exercise, of 95.9±7.8 beats min^−1^ (Table 1, Fig. 2A). Mean minimum heart rates of 74.0±2.8 beats min^−1^ were recorded for birds at-rest for long periods (in excess of five hours). Severe hypoxia had no significant effect on the mean rate at rest (95.2±6.1 beats min^−1^). However, when running at their maximum sustainable speed, heart rate increased to 385.4±5.5 beats min^−1^ during normoxia and to a significantly higher value of 453.2±15.0 beats min^−1^ during severe hypoxia (Table 1, Fig. 2A). Heart rates during running were significantly higher after birds were implanted with heart rate loggers but values at rest were unaffected. Heart rates during running were higher again after implantation of cannulae into the blood vessels while heart rates at rest were significantly lower (Table 2).

**Table 1:** Cardiovascular variables and blood gas data for bar-headed geese at rest and during exercise in normoxia and severe hypoxia (7% O_2_).

	NORMOXIA ( 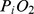 = 21.22 kPa)		HYPOXIA ( 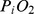 = 7.09 kPa)	
Variable	At rest	Exercise	At rest	Exercise
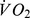 (mL O_2_.min^−1^ .kg^−1^)	19.0±1.3 (7)	43.0±2.7 (7)	14.1 _EST_ (*RER = 0.81*)	40.1 _EST_ (*RER = 0.81*)
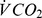 (mL CO_2_.min^−1^ .kg^−1^)	15.5±1.8 (7)	34.7±3.0 (7)	11.4±0.7 (7)	32.5±0.9 (7)
Heart rate 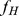 (beats.min^−1^)	95.9±7.8 (5)	385.4±5.5 (5)	95.2±6.1 (6)	453.2±15.0 (5)**
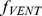 (breaths.min^−1^)	17.3±5.8 (4)	93.8±16.6 (4)	19.7±6.0 (4)	88.8±7.1 (4)
*O_2_ content (mL.dL^−1^)*				
Arterial 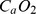	19.1±0.8 (6)	17.7±0.7 (6)	11.2±1.0 (6)**	14.2±1.3 (6)
Mixed venous 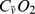	12.7±2.5 (5)	8.9±0.2 (4)*	6.1±1.1 (4)**	5.9±0.7 (5)**
Arterio-Venous difference	5.9 (5)	9.3 (4)	5.4 (4)	9.5 (5)
Estimated 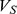 (mL)	7.8	2.8	6.4	2.2
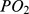 (*kPa*)				
Arterial ( 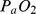 )	13.7±0.04 (7)	14.1±0.06 (6)	5.2±0.03 (7)**	6.3±0.06 (6)**
Mixed venous ( 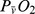 )	7.0±0.06 (6)	5.4±0.05 (5)*	3.7±0.05 (5)**	3.7±0.06 (4)**
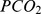 (*kPa*)				
Arterial ( 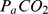 )	5.4±0.2 (6)	4.3±0.4 (6)*	3.2±0.1 (6)**	1.9±0.1 (6)*,**
Mixed venous ( 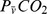 )	6.6±0.4 (5)	6.0±0.4 (5)	3.6±0.1 (5)**	2.6±0.1 (4)*,**
*Lactate* (*mmol.L* ^−1^)				
Arterial	1.1±0.1 (7)	3.8±0.6 (5)	1.5±0.2 (7)	9.0±1.9 (5)*,**
Mixed venous	1.2±0.2 (5)	3.3±0.3 (4)*	1.9±0.4 (5)**	10.0±1.4 (4)*,**
*Haematocrit (%)*				
Arterial	40.1±0.7 (8)	39.3±0.7 (6)	38.5±1.2 (8)	39.5±1.5 (6)
Mixed venous	41.4±0.5 (6)	40.4±0.6 (5)	38.7±1.9 (5)	39.0±2.5 (5)
*Haemoglobin* (*g.dL* ^−1^)				
Arterial	16.5±0.2 (8)	16.0±0.3 (6)	16.9±0.3 (8)	15.9±0.4 (6)
Mixed venous	17.3±0.8 (6)	16.3±0.1 (5)	17.2±1.0 (4)	16.1±0.9 (5)

It was not possible to measure all variables in a single experiment, thus 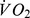 and 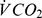 were measured simultaneously, 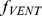 in a second experiment and 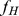 and blood gas variables in a third experiment. Data are mean ± standard error (n); Mass specific values shown for study individuals (mean 2.33 kg, range 2.15 to 2.50 kg); O_2_ content shown as mL O_2_ per dL of blood, haematocrit as percent of packed cells;

*mean different from value at rest,

**mean different from normoxic value.

**Table 2:** Heart rate response to increasing instrumentation for bar-headed geese.

Heart rate (beats.min^−1^)	No logger	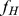 logger	cannulated
*Normoxia*			
At rest	156.5±7.6	138.9±6.6	95.9±7.8
Exercise	278.6±16.1 ^a, b^	348.3±13.4 ^a, c^	385.4±5.5 ^b, c^
*Hypoxia*			
At rest	198.6±5.8 ^d^	173.3±18.4 ^e^	95.2±6.1 ^f,e^
Exercise	394.8±17.8 ^f, g^	466.4±7.6 ^f^	453.2±15.0 ^g^

Paired t-tests p<0.05.

a, b, c, d, e, f, g , treatments represented by different letters.

#### ii) Ventilation rate

In normoxia, bar-headed geese at rest breathed at a mean frequency of 17.3 breaths.min^−1^ (range 11.5 to 29.0, 5.8 s.e.m.), increasing this rate while running to 93.8 breaths.min^−1^ (16.6 s.e.m., maximum 124.0). At rest, bar-headed geese did not alter their breathing frequency in severe hypoxia from that observed in normoxia (19.7 breaths.min^−1^, range 12 to 37.5, 6.0 s.e.m.). Equally, while running, their breathing frequency remained unchanged between normoxia and hypoxia (rate in hypoxia: 88.8 breaths.min^−1^, range 76.5 to 108.3, 7.1 s.e.m.).

#### iii) Rate of O_2_ consumption and CO_2_ production

When at rest in normoxia, bar-headed geese with implanted data loggers had mean 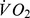 of 19.0 mL.min^−1^.kg^−1^ (±1.3) and 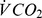 of 15.5 mL.min^−1^.kg^−1^ (±1.8; Table 1, Fig. 2B). While running in normoxia, geese had mean 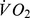 of 43.0 mL.min^−1^.kg^−1^ (±2.7) and mean 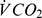 of 34.7 mL.min^−1^.kg^−1^ (±3.0; Fig. 2B). While it was not possible to measure 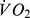 during hypoxia, 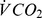 could be measured because baseline incurrent values should have been <0.01% CO_2_. 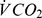 in hypoxia was not different to values recorded in normoxia with mean 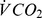 for geese at rest of 11.4 mL.min^−1^.kg^−1^ (±0.7) and 32.5 mL.min^−1^.kg^−1^ (±0.9) for running geese (Fig. 2C). 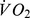 in hypoxia was estimated from the values for 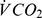 (assuming no change in RER between normoxia and hypoxia), and was found to be similar to those measured in normoxia both at rest and during exercise (Table 1). We reiterate that 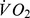
during running exercise in the present study is much lower than the rate of oxygen consumption measured during flight [17] but was associated with the greatest speed that the geese were able to maintain.

#### iv) Acceleration

The physical work carried out by bar-headed geese (as measured using VeDBA) during running in normoxia and severe hypoxia was unchanged (t-test; 0.68±0.04 s.e.m. versus 0.72 g±0.04 s.e.m.).

#### v) Blood gas variables

**Figure 3:**
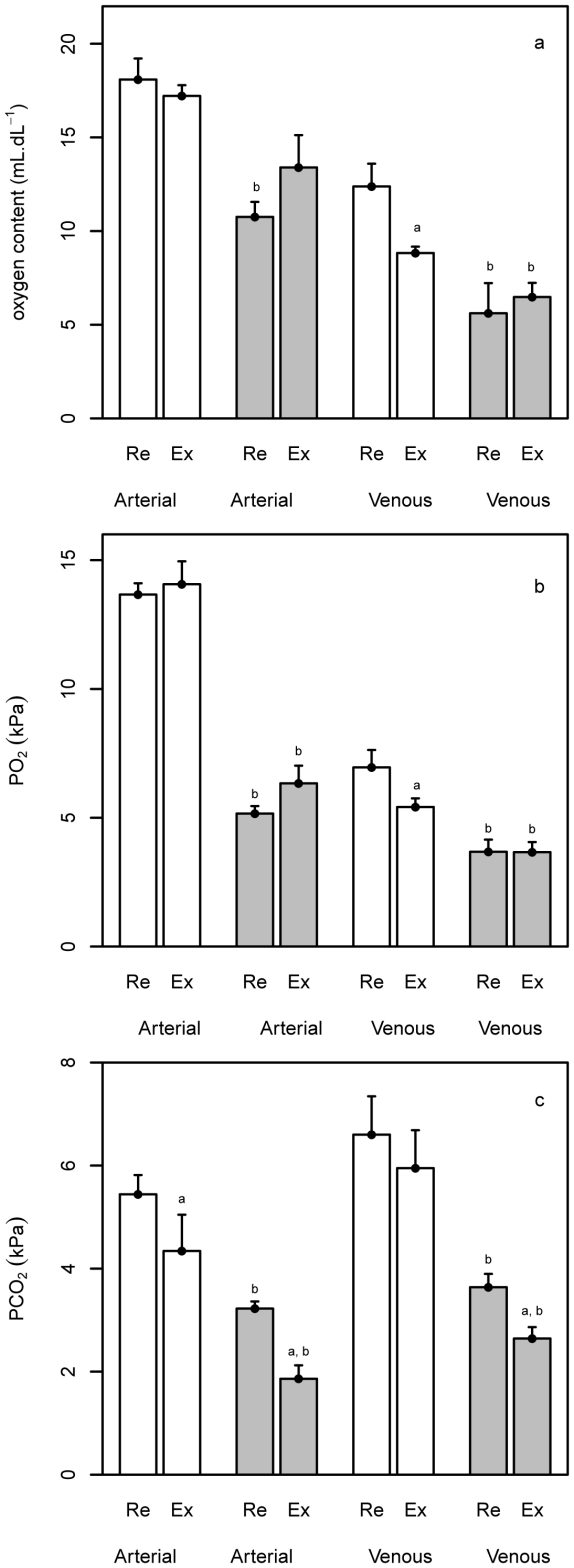
The effect of exercise and hypoxia on blood gases. Bar plots showing the effect of exercise and hypoxia on arterial and mixed venous (a) oxygen content, (b) 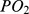 (c) and 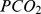 for bar-headed geese. Values at rest (Re) and during running (Ex) are shown for normoxia (white bars) and severe hypoxia (7% O_2_; grey bars). Bars show mean values, error bars show 1 s.e.m., a =  mean values are significantly different at rest, b =  mean values are significantly different to normoxia, where p<0.05.

**Figure 4:**
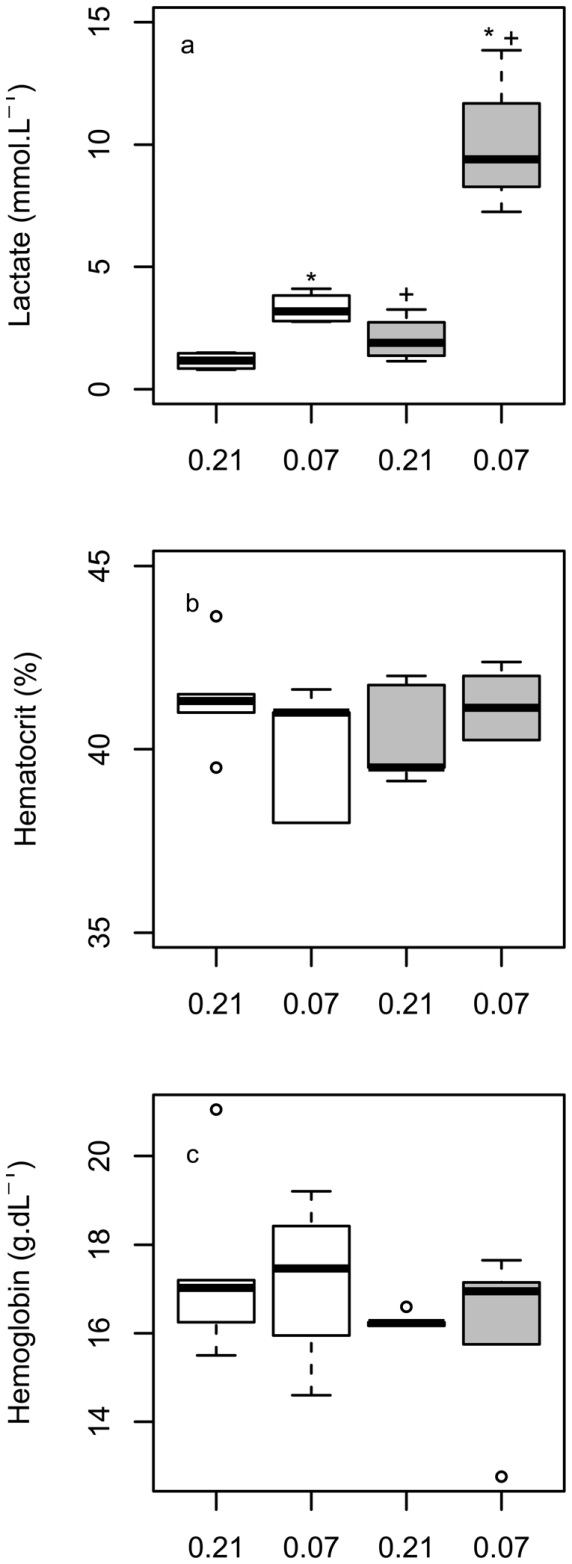
The effect of exercise and hypoxia on lactate, haematocrit and haemoglobin. Box plots showing mixed venous (a) blood lactate, (b) haematocrit and (c) haemoglobin for bar-headed geese at rest (white boxes) and during running (grey boxes) in normoxia and hypoxia (fractional concentration of O_2_ indicated on x-axis). * shows values that are significantly different to values at rest, + shows values that are significantly different to normoxia, where p<0.05.

When cannulated bar-headed geese were exercising in normoxia, their mixed venous oxygen content and 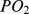 and arterial 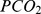 were significantly lower than values at rest while their lactate levels in mixed venous blood increased significantly (Figs. 3 and 4). In severe hypoxia, all blood gas values at rest and during exercise (arterial and mixed venous oxygen content,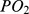 and 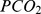) were significantly lower than the values recorded in normoxia (Table 1; Fig. 3). Blood lactate levels were significantly higher in severe hypoxia than in normoxia both while the goose was at rest (mixed venous blood only) and during exercise (arterial and mixed venous blood; Fig. 4, Table 1). Neither exercise nor hypoxia significantly affected blood haematocrit or haemoglobin concentration (Table 1).

**Figure 5:**
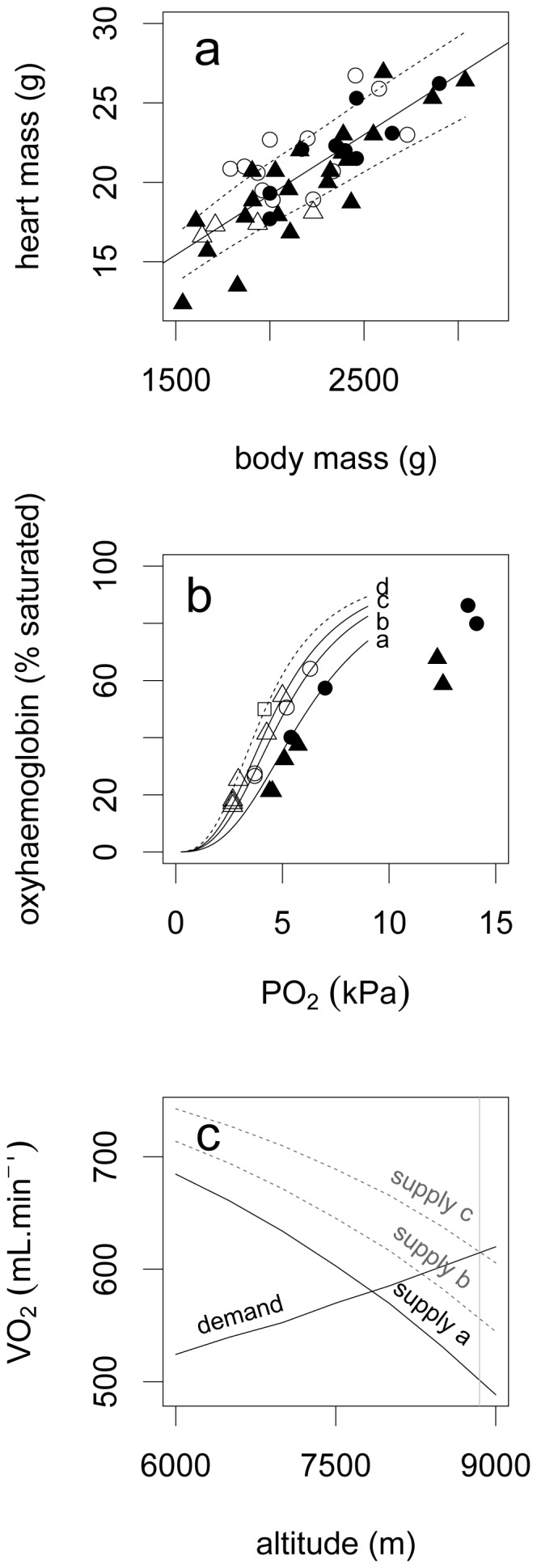
Modelling maximum flight altitude. (a) the relationship between body mass and heart mass for 21 bar-headed geese (circles) and 22 barnacle geese (triangles) from captive collections (white symbols) and the wild (several weeks prior to migration; black symbols), solid line shows linear model (R^2^ = 0.65), dashed lines show confidence interval, *unpublished data*. (b) Oxygen-haemoglobin dissociation curve showing data collected from bar-headed geese in normoxia (black symbols) and hypoxia (white symbols); present study data are shown as circles, data from Fedde et al. [18] as triangles and P_50_ from Meir & Milsom [35] and Petschow et al. [64] (blood in vitro at 41°C and 4% CO_2_, approximating pH 7.4) shown as a white square. Curves are fitted to *in-vivo* data collected in the present study in normoxia (line labelled ‘a’); data collected in the present study in severe hypoxia (line labelled ‘b’) and to data published by Fedde et al. (line labelled ‘c’), excluding four outliers at high 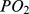. A curve is also fitted to *in-vitro* data published by Petschow et al. [64] and Meir & Milsom [35] (dashed line, labelled ‘d’). (c) Plot showing the modelled increases in 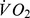 demand (black line) during horizontal flight at high altitude and the reduction in potential O_2_ supply (one black line, two dashed grey lines) by the cardiovascular system. The supply models use the oxygen-haemoglobin dissociation curves in part b (curve a, labelled ‘supply a’; grey dashed curve b, labelled ‘supply b’ and grey dashed curve d labelled ‘supply c’). Grey vertical line approximates the summit of Mount Everest at 8,848 metres. Predicted supply crosses demand at 7,850 m (supply ‘a’), 8,300 m (supply ‘b’) and 8,900 m (supply ‘c’).

We constructed oxygen equilibrium curves during normoxia and severe hypoxia by fitting the above data to the relationship:
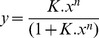
(4)where y is the volume of oxygen, *x* is the 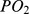 and K and n are constants [34]. Previous studies have determined the value of n (also known as the Hill constant) to be around 2.8 for bar-headed geese [35] and this seemed satisfactory for the data in the present study, with K being 0.01 in hypoxia and 0.006 in normoxia. The partial pressure of oxygen at which the haemoglobin is 50% saturated (*P*_50_) was shifted to the left during hypoxic exercise, from 6.22 kPa to 5.18 kPa in normoxia and hypoxia (Fig. 5B), respectively (assuming that each gram of haemoglobin can carry 1.34 mL O_2_; Hüfner's constant).

### 2. Barnacle geese

#### i) Heart rate

Barnacle geese had mean heart rate at rest, prior to exercise, of 171.4±10.9 beats.min^−1^, which did not increase in moderate or severe hypoxia (139.3±11.7 in moderate hypoxia and 190.6±14.5 during severe hypoxia). Mean minimum heart rates of 81±2.8 beats min^−1^ were recorded for barnacle geese while at rest for long periods in normoxia (in excess of five hours). Heart rate increased during running in moderate hypoxia compared to normoxia (393.9 beats.min^−1^±8.8 in hypoxia vs. 291.9±13.9 in normoxia) but barnacle geese were unable to run for more than four minutes in severe hypoxia, and could only increase mean 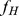 to 316.0±10.3 beats.min^−1^.

#### ii) Rate of O_2_ consumption and CO_2_ production

Mean 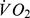 for barnacle geese at rest was 22.7 mL.min^−1^.kg^−1^ (±3.4) and 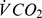 was 17.6 mL.min^−1^ (±3.0; Table 3). While running in normoxia, barnacle geese had mean 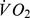 of 47.6 mL.min^−1^.kg^−1^ (±2.7) and 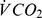 of 39.7 mL.min^−1^.kg^−1^ (±3.9). 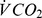 remained unchanged in moderate hypoxia from that observed in normoxia (15.7±2.0 for geese at rest and 38.4±4.9 for running geese; Table 3).

**Table 3:** Cardiovascular variables (heart rate, rate of oxygen consumption, rate of carbon dioxide production and estimated cardiac output) and accelerometer derived VeDBA (see text) for bar-headed geese and barnacle geese at rest and while exercising in normoxia, moderate hypoxia (10.5% O_2_) and severe hypoxia (7% O_2_).

	Bar-headed geese		Barnacle geese	
Variable	At rest	Exercise	At rest	Exercise
**NORMOXIA**				
Heart rate (beats.min^−1^)	156.5±7.6 (7)	278.6±16.1 (7)	171.4±10.9 (10)	291.9±13.9 (10)
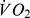 (mL O_2_.min^−1^.kg^−1^)	19.0±1.3 (7)	43.0±2.7* (7)	22.7±3.4 (7)	47.6±2.7* (7)
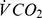 (mL CO_2_.min^−1^.kg^−1^)	15.5±1.8 (7)	34.7±3.0* (7)	17.6±3.0 (7)	39.7±3.9* (7)
VeDBA (G)	0±0 (7)	0.68±0.03 (7)	0±0 (9)	0.66±0.02 (9)
**MODERATE HYPOXIA**				
Heart rate (beats.min^−1^)	183.0±0.8 (7)	395.0±1.1 (7)	139.3±11.7 (10)	393.9±8.8* (10)
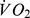 (mL O_2_.min^−1^.kg^−1^)	N/A	N/A	N/A	N/A
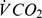 (mL CO_2_.min^−1^.kg^−1^)	13.3±1.7 (7)	38.8±3.3 (7)	15.7±2.0 (7)	38.4±4.9 (7)
VeDBA (G)	N/A	N/A	0±0 (9)	0.66±0.02 (9)
**SEVERE HYPOXIA**				
Heart rate (beats.min^−1^)	198.6±5.8 (7)	394.8±17.8 (7)	190.6±14.5 (6)	316.0±10.3* (6)
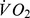 (mL O_2_.min^−1^.kg^−1^)	N/A	N/A	N/A	N/A
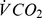 (mL CO_2_.min^−1^.kg^−1^)	11.4±0.7 (6)	32.5±0.9 (7)	N/A	N/A
VeDBA (G)	0±0 (7)	0.72±0.04 (7)	N/A	N/A

Data are mean ± standard error.

*Significantly different from normoxia. N/A data variable not measured. Heart rate for bar-headed geese shown for geese without implanted heart rate loggers or cannulae to facilitate comparison with barnacle geese.

#### iii) Acceleration

The physical work carried out (as measured using VeDBA) during running in normoxia and hypoxia was not different (0.66±0.02 s.e.m. versus 0.66 g±0.02 s.e.m.).

### 3. Modelling maximum flight altitude of bar-headed geese

It was not possible to provide reliable measures for all the Fick equation variables in a single experiment. Therefore, the blood gas data on 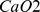 and 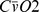 were taken from cannulated geese in both normoxia and hypoxia, along with values for heart rate (Table 1) and the respirometry values for 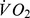 taken from birds with implanted data loggers in normoxia, as there was no difference in the values for VeDBA or for 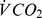 between geese in normoxia and hypoxia, so that 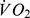 is likely to have been very similar (see also discussion of lactate production, below). When these values are used to estimate mean 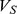 there is a difference between resting values in both normoxia and hypoxia (3.35 mL kg^−1^ and 2.75 mL kg^−1^) compared to those calculated for running geese (1.2 mL kg^−1^ and 0.94 mL kg^−1^; Table 1).

For modelling of the potential maximum rate of oxygen consumption during flight (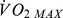) it was thus necessary to combine previously published values. As pointed out in Bishop et al. [8] it is not possible to achieve the relatively high values of 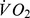 required for flight if the 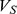 maintained during flight is lower than that measured at rest. In order to estimate maximum 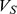 in a wild bird it was first necessary to estimate the likely heart mass for a wild migratory bar-headed goose. Overall, the heart mass when expressed as a proportion of body mass for both captive and wild bar-headed geese and barnacle geese is relatively conserved between 0.9 and 1.1% (data from the present study and Bishop *et al*. unpublished data; Fig. 5A). Thus a hypothetical pre-migratory wild bar-headed goose of 2.82 kg [17] would have an estimated heart mass of 28.2 g. Cannulated bar-headed geese at rest in the present study had a mean calculated 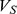 of 7.1 mL and an average heart mass of 21.3 g. Thus, assuming linearity, wild bar-headed geese with a heart mass of 28.2 g would have an estimated mean maximum stroke volume (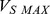) of 9.4 mL.

Geese flying in a wind tunnel sustain values of around 480 beats min^−1^ for 10 minutes (47). We multiplied this by the estimated maximum *V*_S_, to estimate a maximum sustainable cardiac output (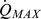) of 4.5 L min^−1^. Substituting into the Fick equation for the convection of blood, along with the mean measured 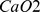 of 0.184 and literature value for mean minimum 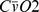
of 0.038, we derive a 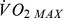 at sea level of around 659 mL min^−1^, which we shall call ‘*supply_SL_*’. Similarly, we can use the measured oxygen saturation curve for the geese running in severe hypoxia to estimate *supply*_HA_ available to exercising bar-headed geese at altitudes potentially encountered by wild geese flying over the Himalayan Mountains and Tibetan-Qinghai Plateau (Fig. 5C). A comparison can then be made between *supply*_HA_ and *demand*_HA_. For example, at 8,500 m *supply*_HA_ is predicted to have fallen to only 531 mL.min^−1^, while *demand*_HA_ is 603 mL.min^−1^. Thus at 8,500 m altitude, *demand*_HA_ is predicted to be greater than *supply*_HA_ and sustained aerobic flight should not be possible. The results of this modelling approach suggest that *supply*_HA_ would meet *demand*_HA_ only until around 7,850 m altitude (Fig. 5C). This is the predicted maximum height to which bar-headed geese might be expected to be found flying aerobically and without wind assistance.

## Discussion

### (i) Comparing bar-headed geese and barnacle geese

The results of our study suggest that bar-headed geese have an extraordinary capacity to perform running aerobic exercise at simulated high altitudes equivalent to the top of Mount Everest, a feat that is not exhibited by lowland living barnacle geese. Although 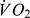
during running was much reduced compared with flight [17], this result was expected as the leg musculature of ducks and geese is smaller than the flight muscle mass [23]. In severe atmospheric hypoxia of 7% oxygen, bar-headed geese were able to run for 15 minutes at the same maximum speed as during normoxia and while maintaining relatively high heart rates, similar to those normally associated with powered flight [17]. This supports the hypothesis that their cardiac muscle should be capable of maintaining high heart rates even while flying in severe hypoxia. In conditions of moderate hypoxia (10.5% atmospheric oxygen), lowland migrating barnacle geese were able to maintain exercise for 15 minutes, with heart rates and 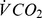 similar to those of bar-headed geese in the same conditions. In contrast to bar-headed geese, however, barnacle geese were unable to run for 15 minutes under conditions of severe hypoxia (7% atmospheric oxygen). What is more, maximal heart rates for the short periods of running they did manage in severe hypoxia were comparatively low, despite the fact that running exercise requires a small proportion of the total aerobic scope exhibited during flight [17]. In humans, peak heart rates are reduced as the level of hypoxia increases [36], which is thought to be evidence for the function of a complex central governor that acts to protect the heart from cardiac ischemia [37]. The hypothesis of a central governor that acts to protect the cardiac and brain oxygenation, by reducing peripheral locomotor muscle activity and decreasing the work of the heart, would be consistent with the observation that the barnacle goose must activate such a mechanism at a lower altitude to that of the bar-headed goose.

This is likely to be because barnacle geese lack some of the adaptations for high altitude that bar-headed geese are known to exhibit. For example, bar-headed geese have modified haemoglobin which improves relative oxygen saturation at lower values of 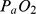, one third higher capillary density in cardiac muscle than barnacle geese [15], [38], a higher capillary to fibre ratio in their flight muscles than barnacle geese [39] and almost 55% greater capillary density than Canada geese in the leg muscles [40]. These adaptations should lead to enhanced maximum O_2_ delivery to the cardiac and locomotory tissue by bar-headed geese relative to barnacle geese. Barnacle geese should not be subject to strong selection for such characters, given that they make the majority of their annual migration over coastal land and seas, and are not known to fly at high altitudes [4]. The high altitude migration of bar-headed geese, instead, has likely selected for physiological adaptations from the cellular [41] to gross morphological level [42] to improve oxygen intake at reduced 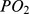 [9]–[11]. However, this is the first time, to the authors' knowledge, that the exercise performance of bar-headed geese in low-oxygen conditions has been demonstrated to be superior to that of other, similar, waterfowl. This supports the suggestion that bar-headed geese are particularly well adapted, compared to other waterfowl, for high altitude flight. Similarly, high altitude native humans resident at 4,350 m show little reduction in cardiac output or 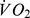 when undergoing maximum exercise tests at altitude compared to sea-level [43], while sea-level natives show a much larger reduction in both variables [44].

Blood gas results were generally comparable to those recorded from previous studies and typically differed by less than ten per cent from those of Fedde et al. [18]. The present study reported the second highest values for 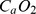 and 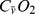 recorded for bar-headed geese to date, both at rest and during exercise in normoxia and hypoxia (n = 5 studies in total). Haemoglobin concentrations were higher in the present study (mean 16.49 g.dL^−1^) than those reported in Black et al. [16] but similar to those reported in Meir & Milsom [35] (17.1 g.dL^−1^). Likewise, the present study had the highest and 2^nd^ highest recorded values for 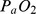 and 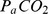, respectively, [45] and higher 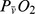 and 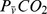 [16], [18], [45], with the exception of one study in which 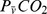 was higher [12]. Our values of 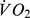 in normoxia were, on average, slightly higher than previously recorded values [17], [46]–[49], but geese in the present study ran faster (up to 1.17 m.sec^−1^). Likewise, heart rates recorded in the present study were slightly higher than those reported in most other studies [17], with heart rates up to 498 beats.min^−1^ for one individual during exercise in hypoxia.

### (ii) Comparison with data in Fedde et al. [18]

The present results suggest an alternative interpretation for the aerobic cost of running in hypoxia than do Fedde et al. [18]. The geese used by Fedde et al. [18] were wearing respirometry face masks (as opposed to being enclosed in a large respirometer in the present study), which may have been stressful and could have contributed to their lower 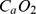, and ventilation frequency, four times higher arterial blood lactate value (when resting in hypoxia) and 2.5-fold higher heart rate at resting. The latter is particularly important as it will have a significant impact on the derivation of *V*_S_. Additionally, Fedde et al. [18] reported more than 50% lower 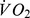 during exercise in severe hypoxia than in normoxia and, after 6 minutes of exercise, they were not significantly higher than values recorded during rest. This result seems highly improbable, given that the biomechanical cost of running was not changed (as indicated by the similar values of VeDBA in the present study), and this would require that 100% of the additional energy requirement for running in hypoxia was met by anaerobic energy sources over a 15 minute period. We suggest that this was unlikely to have been the case, as lactate levels would have had to rise at a rate equivalent to 1 mmol of lactate per 3 mL O_2_.kg^−1^.min^−1^ [50], [51], yielding a minimum total of 48.5 mmol.L^−1^ after only 6.5 minutes of exercise (i.e. 22.4 mL O_2_.min^−1^ kg^−1^/3 * 6.5 minutes). However, the authors recorded a value of only 11.7 mmol.L^−1^ of lactate. A similar calculation for the present study would have yielded lactate levels of 107.4 mmol.L^−1^ at the end of the 15 minute exercise period, far higher than maximum values reported for exercising humans and other mammals [50], [52], [53]. Based on the lactate values reported in Fedde et al. [18], and the similar values recorded during exercise in the present study, at least 90% of the overall costs incurred while running in severe hypoxia in he present study were likely to have been met aerobically (lactate accumulated to 10 mmol.L^−1^ after exercising for six minutes in the present study, a rate of increase in lactate of 1.7 mmol.L^−1^.min^−1^ or only accounting for 5 mL O_2_.kg^−1^.min^−1^). This is in close agreement within the range of lactate values typically reported for animals exercising close to 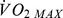 [52], and which is what we would expect from the experimental design. Taken together, this new analysis suggests that the geese in Fedde et al. [18] may have had little scope for the extra cardiovascular demands required during exercise in hypoxia.

### (iii) Modelling maximum flight altitude

Modelling an estimate of maximum flight altitudes, based on the variables of the Fick equation and the oxygen saturation curve for haemoglobin, yields results that are sensitive to the *P*_50_ of the blood during severe hypoxia. Of course, our estimates of oxygen *demand*, derived from measurements taken in a wind tunnel, have a level of uncertainty attached to them. The current state of knowledge of the costs of free flight versus wind tunnel flight does not yet permit an understanding of whether flight in a wind tunnel is less costly than free flight, for example due to wall and ground effects [54], [55], or more challenging [8], [56], for example as demonstrated by lower heart rates in wild migrating birds than in captive birds flying in a wind tunnel [4]. This is an important limitation of the present modelling work.

Overall, it appears unlikely that cardiac output could be much larger than estimated in the present study. The maximum value we calculated is approximately 50% higher than the highest cardiac output previously measured in bar-headed geese during severe hypoxia [16]. Maximal *f*_H_ and *V*_s_ have been well described for mammals and studies have suggested that birds should have reasonably similar maxima for a given heart and body mass [57]–[59]. Thus, the most significant potential uncertainty in the present analysis is associated with the reliance on the blood parameters and haemoglobin-oxygen saturation curve of the blood. However, the pathway for oxygen during running is exactly the same as that for flight, apart from the final unloading phase of oxygen at the smaller musculature of the hind limbs, rather than the forelimbs. It may seem reasonable that wild migratory bar-headed geese could maintain slightly higher oxygen content of the arterial blood than we measured in the present study by increasing the haemoglobin levels prior to migration [60]–[62]. It may also be possible that under extreme circumstances geese could deplete venous oxygen stores even more than suggested, for example, it has been shown that thoroughbred racehorses exercising on a treadmill depleted mixed venous 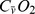 to almost 2 mL.dL^−1^ [63] under maximum exercise conditions. However, data describing whether these physiological adaptations occur in the wild are currently lacking and captive bar-headed geese exposed to hypoxia do not increase their haemoglobin concentration [16].

One of the most effective ways for bar-headed geese to increase their oxygen loading of the arterial blood at high altitudes would be to directly modulate the functioning of the oxygen-haemoglobin dissociation curve at the blood-gas interface of the lung. In this context, oxygen affinity of the blood of bar-headed geese has been shown to be sensitive to both pH and temperature [35], but without interaction. Certainly, blood at the gas exchange interface of the lung is always likely to be more alkaline than that at the core of the body due to the rapid diffusion of CO_2_ into the lungs, and this will stimulate a Bohr shift of the oxygen loading curve to the left and enhance 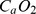 (at any given physiologically relevant blood temperature). However, in the present state of knowledge, it is not possible to ascertain how much higher the pH of the lung capillary blood might be during wild migratory flights, as against those experienced by captive birds running on a treadmill. Fedde *et al*. [18] measured a pH of 7.53 and a body temperature of 41.5°C during hypoxic running and, at least the latter, seems quite appropriate for a bird during flapping flight. Oxygen loading could also be enhanced if ventilation and pulmonary oxygen diffusion were able to reduce the inspired-arterial O_2_ difference by a greater magnitude than estimated in our modelling.

While the modelling analysis, based on data from the present study, indicates a maximum aerobic flight altitude of around 7,850 m (Fig. 5C, line a), utilising the oxygen saturation curve determined by Fedde et al. [18] in hypoxia yields a maximum altitude prediction of 8,300 m. While these two studies are based on *in vivo* samples taken from exercising birds, two *in vitro* studies found an even lower value for *P*_50_ in bar-headed goose blood [35], [64]. If wild geese do indeed have similar oxygen saturation curve characteristics to these *in vitro* studies, the predicted maximum aerobic altitude can be increased to almost 8,900 m. However, if geese flew at this altitude, it would have to be at 100% of their estimated 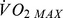 to sustain horizontal flight. This compares with elite ultra-marathon runners who show a clear decline in the fraction of 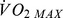 they can sustain during prolonged exercise and were predicted to be able to maintain 87.5% of 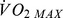 over a 1 hour period [65]. This might indicate that, even if capable of level flight at such altitudes, sustained flight may be questionable. In addition, our maximum altitude modelling does not take into account the considerable additional cost required to climb to high altitude in still air conditions, which for a 2.82 kg goose could be around 108 mL O_2_ min^−1^ at a climb rate of 0.3 m s^−1^, in addition to the costs of level flapping flight [33]. Thus, our estimates of 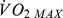 would suggest that it might only be possible for a bar-headed goose to employ climbing flight, in still air, until 6,800 m (Fig. 5C, line a), or up to 7,850 m with maximum oxyhaemoglobin loading characteristics (Fig. 5C, line c), or slightly higher if at a reduced climb rate. These heights are consistent with observations from tracking data; the highest altitude directly recorded from a bar-headed goose crossing the Himalayas so far is 7,290 m above sea level (but such altitudes are rare, with 95% of GPS recorded altitudes below 5,784 m [3].

Regardless of the absolute limits to the rates of oxygen consumption of bar-headed geese, the environment will also impact on their ability to fly to high altitudes. Additional height may be achieved with wind assistance in the mountains (orographic lift), or at night when the density altitude may be lower, or with a combination of these factors [2], but is apparently rarely the case [2], [3]. The ‘density altitude’ (the corresponding altitude in the international standard atmosphere at which the observed conditions occur) experienced by migrating bar-headed geese can also vary considerably with ambient temperature, such that travelling at night when conditions are colder and air is denser, could increase the maximum altitude at which flight is possible by several hundred metres [2]. This is a phenomenon that is exploited by human mountaineers in the Himalayas [66]. While it is possible that incremental changes of this type may facilitate marginal increases in 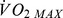, or influence air density and air currents such that higher altitude aerobic flights could be possible, overall, this analysis suggests that such high flights might be extremely challenging and would likely be rare if they take these birds to the edge of their capabilities.

## Conclusion

In the present study we have shown that bar-headed geese are capable of maximum sustained running exercise in conditions of hypoxia similar to the summit of Mount Everest. We have also shown that barnacle geese, a lowland species from the same *Anserini* tribe, are not. Notwithstanding the multitude of adaptations for high altitude exercise that are known in bar-headed geese [9]–[11], the primary difference appears to be due to enhanced cardiac performance in hypoxic conditions in the bar-headed goose relative to the barnacle goose. Bar-headed geese can maintain heart rates, even in severe hypoxia, similar to those recorded in powered flight [17]. While there may be some uncertainties in the absolute values used in our modelling exercise, we suggest that unassisted, level, aerobic powered flight (i.e. without wind assistance) should be possible for geese with a pH of around 7.5 and body temperature of 41°C up to almost 8,000 metres altitude. Flight higher than this, and above the peak of Mount Everest (8,848 m), would require oxygen supply to increase by an additional 25% but might be possible with a *P*_50_ value determined from *in vitro* blood analysis. Even then, geese would require additional power to climb unaided to such altitudes. Our results suggest that bar-headed geese should not generally be expected to be found migrating over the highest summits of the Himalayan Mountains, unless aerobic flight can be supported by some kind of climatological assistance and/or flight-enhanced gas exchange.

## Supporting Information

Figure S1
**Schematic diagram of the process used to model maximum flight altitude.** “Demand” is modelled from 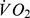 collected from bar-headed geese in a wind tunnel [17] multiplied by the additional power requirements with altitude from a flight biomechanical model [33]. “Supply” is modelled from components of the Fick equation, indicated, generating a distribution of 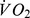 values for 500 metre altitudinal increments.(PDF)

## References

[1] Bishop M, Yanling S, Zhouma C, Binyuan G (1997) Bar-headed geese *Anser indicus* wintering in south-central Tibet. Wildfowl 48: 118–126.

[2] Hawkes LA, Balachandran S, Batbayar N, Butler PJ, Frappell PB, et al (2011) The trans-Himalayan flights of bar-headed geese (*Anser indicus*). Proceedings of the National Academy of Sciences 108: 9516–9519. 10.1073/pnas.1017295108 PMC3111297 21628594

[3] Hawkes LA, Balachandran S, Batbayar N, Butler PJ, Chua B, et al (2013) The paradox of extreme high altitude migration in bar-headed geese *Anser indicus* . Proceedings of the Royal Society B 280: 20122114. 23118436 10.1098/rspb.2012.2114 PMC3574432

[4] Butler PJ, Woakes AJ, Bishop CM (1998) Behaviour and physiology of Svalbard barnacle geese *Branta leucopsis* during their autumn migration. Journal of Avian Biology 29: 536–545.

[5] Portugal SJ, Green JA, White CR, Guillemette M, Butler PJ (2011) Wild geese do not increase flight behaviour prior to migration. Biology Letters 8: 469–472. 22090201 10.1098/rsbl.2011.0975 PMC3367731

[6] Prop J, Black JM, Shimmings P (2003) Travel schedules to the high arctic: barnacle geese trade-off the timing of migration with accumulation of fat deposits. Oikos 103: 403–414.

[7] Eichhorn G, Drent RH, Stahl J, Leito A, Alerstam T (2009) Skipping the Baltic: the emergence of a dichotomy of alternative spring migration strategies in Russian barnacle geese. Journal of Animal Ecology 78: 63–72. 19120596 10.1111/j.1365-2656.2008.01485.x

[8] Bishop CM, Ward S, Woakes AJ, Butler PJ (2002) The energetics of barnacle geese (*Branta leucopsis*) flying in captive and wild conditions. Comparative Biochemistry and Physiology - Part A: Molecular & Integrative Physiology 133: 225–237. 10.1016/s1095-6433(02)00157-5 12208297

[9] Butler PJ (2010) High fliers: The physiology of bar-headed geese. Comparative Biochemistry and Physiology - Part A: Molecular & Integrative Physiology 156: 325–329. 10.1016/j.cbpa.2010.01.016 20116442

[10] Scott GR, Milsom WK (2006) Flying high: A theoretical analysis of the factors limiting exercise performance in birds at altitude. Respiratory Physiology & Neurobiology 154: 284–301. 16563881 10.1016/j.resp.2006.02.012

[11] Faraci FM (1991) Adaptations to hypoxia in birds: how to fly high. Annual Review of Physiology 53: 59–70. 10.1146/annurev.ph.53.030191.000423 2042973

[12] Scott GR, Milsom WK (2007) Control of breathing and adaptation to high altitude in the bar-headed goose. American Journal of Physiology - Regulatory, Integrative and Comparative Physiology 293: R379–R391. 10.1152/ajpregu.00161.2007 17491113

[13] Faraci FM, Kilgore Jr DL, Fedde MR (1985) Blood flow distribution during hypocapnic hypoxia in pekin ducks and bar-headed geese. Respiration Physiology 61: 21–30. 3929351 10.1016/0034-5687(85)90025-8

[14] Faraci FM, Kilgore DL, Fedde MR (1984) Attenuated pulmonary pressor response to hypoxia in bar-headed geese. American Journal of Physiology - Regulatory, Integrative and Comparative Physiology 247: R402–R403. 10.1152/ajpregu.1984.247.2.R402 6465351

[15] Faraci FM, Kilgore DL, Fedde MR (1984) Oxygen delivery to the heart and brain during hypoxia: Pekin duck vs. bar-headed goose. American Journal of Physiology - Regulatory, Integrative and Comparative Physiology 247: R69–R75. 10.1152/ajpregu.1984.247.1.R69 6742237

[16] Black CP, Tenney SM (1980) Oxygen transport during progressive hypoxia in high-altitude and sea-level waterfowl. Respiration Physiology 39: 217–239. 7375742 10.1016/0034-5687(80)90046-8

[17] Ward S, Bishop CM, Woakes AJ, Butler PJ (2002) Heart rate and the rate of oxygen consumption of flying and walking barnacle geese (*Branta leucopsis*) and bar-headed geese (*Anser indicus*). Journal of Experimental Biology 205: 3347–3356. 12324544 10.1242/jeb.205.21.3347

[18] Fedde MR, Orr JA, Shams H, Scheid P (1989) Cardiopulmonary function in exercising bar-headed geese during normoxia and hypoxia. Respiration Physiology 77: 239–252. 2506620 10.1016/0034-5687(89)90010-8

[19] Butler PJ (1991) Exercise in Birds. Journal of Experimental Biology 160: 233–262.

[20] Swan L (1970) Goose of the Himalayas. Natural History 79: 68–75.

[21] Swan LW (1961) Ecology of the high Himalayas. Scientific American 205: 68–78.

[22] Blum A (1998) Annapurna: A Woman's Place: Sierra Book Clubs. 272 p.

[23] Hartman FA, Brownell KA (1961) Lipids in the Locomotor Muscles of Birds. The Condor 63: 403–409.

[24] Stephenson R, Butler PJ, Woakes AJ (1986) Diving behaviour and heart rate in tufted ducks (*Aythya fuligula*). Journal of Experimental Biology 126: 341–359. 3805996 10.1242/jeb.126.1.341

[25] Fedak MA, Rome L, Seeherman HJ (1981) One-step N_2_-dilution technique for calibrating open-circuit VO_2_ measuring systems. Journal of Applied Physiology 51: 772–776. 7327980 10.1152/jappl.1981.51.3.772

[26] Lighton JRB (2008) Measuring metabolic rates: a manual for scientists: Oxford University Press.

[27] Withers PC (1977) Measurement of VO_2_, VCO_2_, and evaporative water loss with a flow-through mask. Journal of Applied Physiology 42: 120–123. 833070 10.1152/jappl.1977.42.1.120

[28] Schippers MP, Ramirez O, Arana M, Pinedo-Bernal P, McClelland GB (2012) Increase in carbohydrate utilization in high-altitude Andean mice. Current Biology 22: 2350–2354. 23219722 10.1016/j.cub.2012.10.043

[29] Woakes AJ, Butler PJ (1986) Respiratory, circulatory and metabolic adjustments during swimming in the tufted duck, *Aythya fuligula* . Journal of Experimental Biology 120: 215–231.

[30] Bond CF, Gilbert PW (1958) Comparative study of blood volume in representative aquatic and nonaquatic birds. American Journal of Physiology 194: 519–521. 13571419 10.1152/ajplegacy.1958.194.3.519

[31] Rodkey FL, Robertson RF, Kim CK (1979) Molar absorbance of cyanmethemoglobin from blood of different animals. American Journal of Veterinary Research 40: 887–888. 475142

[32] Gleiss AC, Wilson RP, Shepard ELC (2011) Making overall dynamic body acceleration work: on the theory of acceleration as a proxy for energy expenditure. Methods in Ecology and Evolution 2: 23–33.

[33] Pennycuick CJ (2008) Modelling the flying bird. Academic Press, London.

[34] Brown WEL, Hill AV (1923) The Oxygen dissociation curve of blood and it's thermodynamical basis. Proceedings of the Royal Society B 94: 297–334.

[35] Meir JU, Milsom WK (2013) High thermal sensitivity of blood enhances oxygen delivery in the high-flying bar-headed goose. The Journal of Experimental Biology 216: 2172–2175. 23470665 10.1242/jeb.085282

[36] Lundby C, Araoz M, van Hall G (2001) Peak heart rate decreases with increasing severity of acute hypoxia. High Altitude Medicine & Biology 2: 369–376. 11682016 10.1089/15270290152608543

[37] Noakes TD, Marino FE (2007) Arterial oxygenation, central motor output and exercise performance in humans. Journal of Physiology 585: 919–921. 17962324 10.1113/jphysiol.2007.145110 PMC2375511

[38] Scott GR (2011) Elevated performance: the unique physiology of birds that fly at high altitudes. The Journal of Experimental Biology 214: 2455–2462. 21753038 10.1242/jeb.052548

[39] Scott GR, Egginton S, Richards JG, Milsom WK (2009) Evolution of muscle phenotype for extreme high altitude flight in the bar-headed goose. Proceedings of the Royal Society B: Biological Sciences 276: 3645–3653. 19640884 10.1098/rspb.2009.0947 PMC2817306

[40] Snyder GK, Byers RL, Kayar SR (1984) Effects of hypoxia on tissue capillarity in geese. Respiration Physiology 58: 151–160. 6522870 10.1016/0034-5687(84)90144-0

[41] Scott GR, Richards JG, Milsom WK (2009) Control of respiration in flight muscle from the high-altitude bar-headed goose and low-altitude birds. American Journal of Physiology - Regulatory, Integrative and Comparative Physiology 297: R1066–R1074. 10.1152/ajpregu.00241.2009 19657102

[42] Lee SY, Scott GR, Milsom WK (2008) Have wing morphology or flight kinematics evolved for extreme high altitude migration in the bar-headed goose? Comparative Biochemistry and Physiology Part C: Toxicology & Pharmacology 148: 324–331. 18635402 10.1016/j.cbpc.2008.05.009

[43] Vogel JA, Hartley LH, Cruz JC (1974) Cardiac output during exercise in altitude natives at sea level and high altitude. Journal of Applied Physiology 36: 173–176. 4590353 10.1152/jappl.1974.36.2.173

[44] Vogel JA, Hartley LH, Cruz JC, Hogan RP (1974) Cardiac output during exercise in sea-level residents at sea level and high altitude. Journal of Applied Physiology 36.. 10.1152/jappl.1974.36.2.169 4590352

[45] Faraci FM, Fedde MR (1986) Regional circulatory responses to hypocapnia and hypercapnia in bar-headed geese. American Journal of Physiology - Regulatory, Integrative and Comparative Physiology 250: R499–R504. 10.1152/ajpregu.1986.250.3.R499 3082220

[46] Tickle PG, Richardson MF, Codd JR (2010) Load carrying during locomotion in the barnacle goose (*Branta leucopsis*): The effect of load placement and size. Comparative Biochemistry and Physiology 156: 309–317. 10.1016/j.cbpa.2010.01.022 20153444

[47] Nudds RL, Gardiner JD, Tickle PG, Codd JR (2010) Energetics and kinematics of walking in the barnacle goose (*Branta leucopsis*). Comparative Biochemistry and Physiology Part A: Molecular & Integrative Physiology 156: 318–324. 10.1016/j.cbpa.2010.01.023 20138237

[48] Portugal SJ, Green JA, Cassey P, Frappell PB, Butler PJ (2009) Predicting the rate of oxygen consumption from heart rate in barnacle geese *Branta leucopsis*: effects of captivity and annual changes in body condition. Journal of Experimental Biology 212: 2941–2948. 19717676 10.1242/jeb.034546

[49] Nolet BA, Butler PJ, Masman D, Woakes AJ (1992) Estimation of Daily Energy Expenditure from Heart Rate and Doubly Labeled Water in Exercising Geese. Physiological Zoology 65: 1188–1216.

[50] di Prampero PE, Ferretti G (1999) The energetics of anaerobic muscle metabolism: a reappraisal of older and recent concepts. Respiration Physiology 118: 103–115. 10647856 10.1016/s0034-5687(99)00083-3

[51] Margaria R, Cerretelli P, diPrampero PE, Massari C, Torelli G (1963) Kinetics and mechanism of oxygen debt contraction in man. Journal of Applied Physiology 18: 371–377. 13932994 10.1152/jappl.1963.18.2.371

[52] Seeherman HJ, Taylor RC, Maloiy GMO, Armstrong RB (1981) Design of the mammalian respiratory system. II. Measuring maximum aerobic capacity. Respiration Physiology 44: 11–23. 7232881 10.1016/0034-5687(81)90074-8

[53] Beneke R (2003) Maximal lactate steady state concentration (MLSS): experimental and modelling approaches. European Journal of Applied Physiology 88: 361–369. 12527964 10.1007/s00421-002-0713-2

[54] Rayner JMV (1991) On the aerodynamics of animal flight in ground effect. Philosophical Transactions of the Royal Society of London Series B: Biological Sciences 334: 119–128.

[55] Rayner JMV (1994) Aerodynamic corrections for the flight of birds and bats in wind tunnels. Journal of Zoology 234: 537–563.

[56] Liechti F, Bruderer L (2002) Wingbeat frequency of barn swallows and house martins: a comparison between free flight and wind tunnel experiments. Journal of Experimental Biology 205: 2461–2467. 12124369 10.1242/jeb.205.16.2461

[57] Bishop CM, Spivey RJ (2013) Integration of exercise response and allometric scaling in endotherms. Journal of Theoretical Biology 323: 11–19. 23328533 10.1016/j.jtbi.2013.01.002

[58] Bishop C, Butler P (1995) Physiological modelling of oxygen consumption in birds during flight. Journal of Experimental Biology 198: 2153–2163. 9320064 10.1242/jeb.198.10.2153

[59] Bishop CM (1999) The maximum oxygen consumption and aerobic scope of birds and mammals: getting to the heart of the matter. Proceedings of the Royal Society B 266: 2275–2281. 10629977 10.1098/rspb.1999.0919 PMC1690458

[60] Verhulst S, Oosterbeek K, Bruinzeel LW (2002) Haematological parameters, mass and moult status in dunlins *Calidris alpina* preparing for spring migration. Avian Science 2: 1–8.

[61] Ruiz GJ, Rosenmann M, Fernando Novoa E (1995) Seasonal changes of blood values in Rufous-collared sparrows from high and low altitude. International Journal of Biometeorology 39: 103–107.

[62] Piersma T, Everaarts JM, Jukema J (1996) Build-up of red blood cells in refuelling bar-tailed godwits in relation to individual migratory quality. The Condor 98: 363–370.

[63] Butler PJ, Woakes AJ, Smale K, Roberts CA, Hillidge CJ, et al (1993) Respiratory and cardiovascular adjustments during exercise of increasing intensity and during recovery in thoroughbred racehorses. Journal of Experimental Biology 179: 159–180. 8340728 10.1242/jeb.179.1.159

[64] Petschow D, Wurdinger I, Baumann R, Duhm J, Braunitzer G, et al (1977) Causes of high blood O_2_ affinity of animals living at high altitude. Journal of Applied Physiology 42: 139–143. 14096 10.1152/jappl.1977.42.2.139

[65] Davies CTM, Thompson MW (1979) Aerobic performance of female marathon and male ultramarathon athletes. European Journal of Applied Physiology and Occupational Physiology 41: 233–245. 499187 10.1007/BF00429740

[66] West JB, Boyer SJ, Graber DJ, Hackett PH, Maret KH, et al (1983) Maximal exercise at extreme altitudes on Mount Everest. Journal of Applied Physiology 55: 688–698. 6415008 10.1152/jappl.1983.55.3.688

