# Supplementary figures and images for: Maximum Running Speed of Captive Bar-Headed Geese Is Unaffected by Severe Hypoxia
Source: PLoS One. 2014 Apr 7;9(4):e94015. doi: 10.1371/journal.pone.0094015 (PMC3977980; doi:10.1371/journal.pone.0094015)

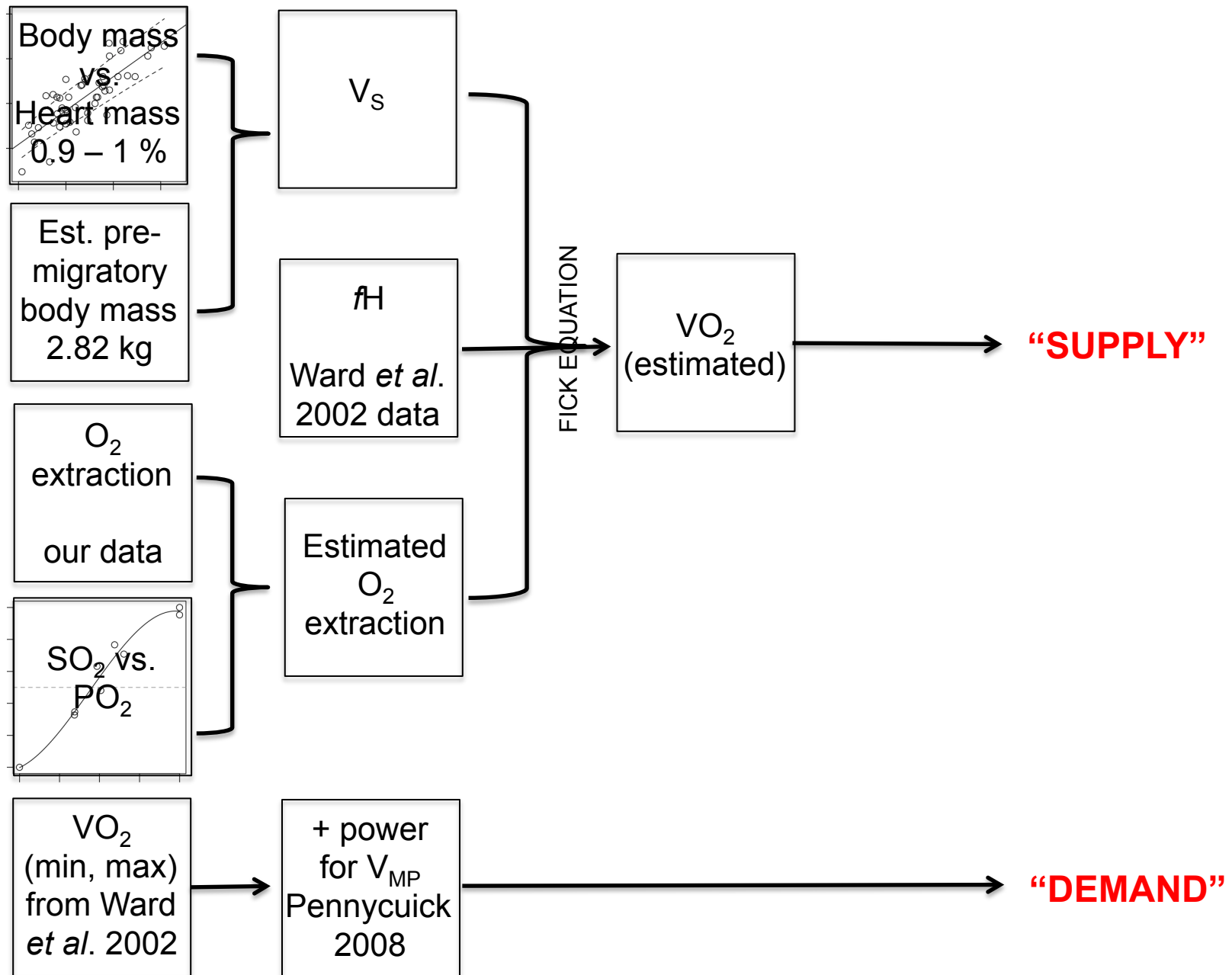

Supplement: Figure S1 — Schematic diagram of the process used to model maximum flight altitude. “Demand” is modelled from collected from bar-headed geese in a wind tunnel [17] multiplied by the additional power requirements with altitude from a flight biomechanical model [33]. “Supply” is modelled from components of the Fick equation, indicated, generating a distribution of values for 500 metre altitudinal increments. (PDF) [file pone.0094015.s001.pdf]
